# Addressing maternal healthcare through demand side financial incentives: experience of Janani Suraksha Yojana program in India

**DOI:** 10.1186/1472-6963-12-319

**Published:** 2012-09-15

**Authors:** Saji S Gopalan, Durairaj Varatharajan

**Affiliations:** 1 The World Bank, 1818 H Street NW, Washington, DC 20433, USA; 2 Department of Health Systems financing, The World Health Organization, 20 Avenue Appia, Geneva, Switzerland

**Keywords:** Maternal healthcare consumption, Out-of-pocket spending, Demand side financing, Financial risk-protection, Low-and-middle income countries, India

## Abstract

**Background:**

Demand side financing (DSF) is a widely employed strategy to enhance utilization of healthcare. The impact of DSF on health care seeking in general and that of maternal care in particular is already known. Yet, its effect on financial access to care, out-of-pocket spending (OOPS) and provider motivations is not considerably established. Without such evidence, DSFs may not be recommendable to build up any sustainable healthcare delivery approach. This study explores the above aspects on India’s Janani Suraksha Yojana (JSY) program.

**Methods:**

This study employed design and was conducted in three districts of Orissa, selected through a three-stage stratified sampling. The quantitative method was used to review the Health Management Information System (HMIS). The qualitative methods included focus groups discussions with beneficiaries (n = 19) and community intermediaries (n = 9), and interviews (n = 7) with Ministry of Health officials. HMIS data enabled to review maternal healthcare utilization. Group discussions and interviews explored the perceived impact of JSY on in-facility delivery, OOPS, healthcare costs, quality of care and performance motivation of community health workers.

**Results:**

The number of institutional deliveries, ante-and post-natal care visits increased after the introduction of JSY with an annual net growth of 18.1%, 3.6% and 5% respectively. The financial incentive provided partial financial risk-protection as it could cover only 25.5% of the maternal healthcare cost of the beneficiaries in rural areas and 14.3% in urban areas. The incentive induced fresh out-of-pocket spending for some mothers and it could not address maternal care requirements comprehensively. An activity-based community worker model was encouraging to augment maternal healthcare consumption. However, the existing level of financial incentives and systemic support were inadequate to motivate the volunteers optimally on their performance.

**Conclusion:**

Demand side financial incentive could enhance financial access to maternal healthcare. However, it did not adequately protect households from financial risks. An effective integration of JSY with similar social protection or financial risk-protection measures may protect mothers substantially from potential out-of-pocket spending. Further, this integrated approach may help upholding more awareness on maternal health rights and entitlements. It can also address maternal health beyond ‘maternal healthcare’ and ensure sustainability through pooled financial and non-financial resources.

## Background

Demand side financial incentive (DSF) is a form of subsidy and it directly provides purchasing ability to consumers on certain publicly provided goods such as health and nutrition [1]. DSF introduces two key changes in the public financing of such goods and services [1]. First, it entitles the government to be a supplier of purchasing power to consumers instead of being a direct service provider. Secondly, it tunes financing as ‘output-based’ than ‘input-based’ and hence links the subsidy or its objective with the beneficiary. DSF’s importance primarily lies on its scope to integrate various human development approaches and advance individual and societal capabilities. Under DSF, overall human development occurs as it addresses populations’ social, environmental and economic risks or vulnerabilities [2].

 In health sector, DSF has a possible role in the delivery of certain sub-optimally and inequitably consumed services (e.g. maternal care) and betterment of unmet health behaviors [3-5]. DSF operates on the principal-agent theory, where the principal (government, donor or community) transfers funds to an agent (consumer) conditional on a defined action [6]. Primarily it caters to under-served areas, populations and services [6]. Its underlying principle is to synergize the supply-and demand-sides through additional demand generation and supply strengthening [4,7]. Thus, DSF links various supply-and demand-side measures (e.g. service provision and community awareness) with differential financing approach [3,7]. It also harnesses the private sector potential and promotes innovative pooling and transfer of funds. As of now, among the various health needs, DSF has predominantly focused on millennium development goals (MDG) [7].

### Demand side financial incentives for maternal healthcare: What is already known?

In many regions and countries (e.g. Latin America, India, Nepal and Bangladesh) the earliest public sector DSF initiatives catered to maternal and child health (MCH) [8-10]. This MCH focus was due to such countries’ persistent need to meet MDGs 4 and 5. Among the variants of DSF, vouchers and conditional cash transfers are more widespread on MCH compared to health insurance. This is because the former appear to be better streamlined to achieve specified outcomes in a given timeframe [2,3,10]. DSF’s design, roll-out and monitoring vary across countries. For instance, the Indian DSF initiatives exhibit less convergence of maternal care with reproductive health services, unlike those many Latin American and African countries.

DSF’s impact on intermediate outcomes such as skilled birth attendance and consumer awareness are considerably documented, though their long-term benefits on maternal health status are awaited [4]. However, the evidence on their contribution to out-of-pocket spending (OOPS) and performance motivation of providers is scanty. Although, they target purchasing power provision to reduce OOPS, the prime focus on achieving the specified health targets derail them from addressing OOPS largely [4]. This may ultimately concern the fulfillment of MDG 1 (i.e. poverty reduction) in many countries, even if they are able to achieve other health related MDGs. Further, without understanding the intricacies of OOPS vis-à-vis DSF, healthcare delivery may not be appropriately organized under DSF. The optimal packaging of incentives for rational consumer choices may not be reasonable without understanding the scope of OOPS under DSF. Many resource constraint settings introduced DSF to enhance skilled birth attendance with the intermediation of community health workers [4]. Without ensuring their performance motivation, the achievement of specific community response and effectiveness of the financial resources employed may not be feasible.

The present study on *Janani Suraksha Yojana* (JSY) program in India explored the following; 1) JSY’s potential to enhance women’s financial access to maternal healthcare, 2) its effect on household out-of-pocket spending (OOPS) on maternal healthcare, and 3) its influence on community health workers’ performance motivation.

## Methods

### Janani Suraksha Yojana: an overview

JSY is a federal government funded nation-wide scheme offering conditional cash transfers for safe motherhood since 2005 [11,12]. JSY was initiated in the milieu of India’s persistently alarming maternal and infant deaths [11-13]. Paid in bank cheques after child birth, the incentive is conditional upon either in-facility delivery or skilled birth attendance [14,15]. Women are encouraged to avail free ante- and post-natal care in public facilities, but no incentive is linked to such care [14,15].

JSY holds different conditions for low-and high-performing states, a classification by Government of India (GOI) based on basic demographic and health indicators [14,15].

 In low-performing states like Orissa, women of all socio-economic and demographic profiles are covered, irrespective of the order of their child birth [14,15]. Upon in-facility delivery, each pregnant woman receives US$ 30.10 in rural areas and US$ 21.50 in urban areas [16]. The incentive for home delivery is US$ 10.75. The community health worker (known as Accredited Social Health Activist or ASHA) receives US$ 7.50 per pregnant woman in rural areas (US$ 4.30 in urban areas) for coordinating ante-and post-natal care and escorting for in-facility delivery. An additional sum of (ANC and PNC) US$ 5.30 is also packaged with ASHA incentives to cover transport costs of mothers. In high-performing states, JSY addresses only socio-economically backward women and each eligible woman receives US$ 15.05 and 12.90 in rural and urban areas respectively [16].

The state governments manage the scheme and empanel healthcare providers, including the private ones, who fulfill certain eligibility criteria [15,17]. JSY’s annual financial allocation increased from US$ 8.2 million to US$ 266.8 million between 2005–06 and 2008–09. The number of beneficiaries increased from 0.73 million to 8·43 million, covering a third of the 26 million child births annually in India during the same period. For the same period, the operational cost per beneficiary had amplified from US$11.24 to US$ 31.65 [17].

### Study setting and sampling

The study was performed in *Orissa,* a socio-economically backward state with 85% rural, 40% poor and 22% indigenous population [18]. Maternal mortality ratio in Orissa is 540 per 100,000 live births (national average 301) and 39% of deliveries are institutional (national average 40.7%) [18]. The study settings were selected through a three-stage stratified random sampling. In the first stage, Orissa was selected among low-performing states. In the second stage, the districts of Gajapati, Nayagarh, and Mayurbhanj were chosen representing the administrative division of the state. Finally, a half of the rural and urban blocks from each district were included in the study, which together had 10% of Orissa’s population.

### Data collection

This study employed a ‘mixed-methods design’ and was carried out in the first half of 2010. The quantitative method was employed to review the Health Management Information System (HMIS) data. The qualitative methods consisted of focus group discussions (FGDs) with mothers (JSY beneficiaries) and ASHAs (JSY intermediaries). Further, there were key-informant interviews with the Ministry of Health (MoH) officials (JSY and healthcare providers). The study also reviewed some reports and policy pronouncements to validate the data gathered from HMIS, FGDs and interviews. The beneficiaries were identified with the help of ASHAs, women’s groups and other community-based entities. The ASHAs were identified through the local MoH program officers.

#### Review of documents and data sources

The indicators on maternal healthcare consumption (i.e. institutional deliveries, ANC and PNC) and outcomes (maternal deaths) from HMIS were compared before (2002–2004) and during the implementation (2005–2010) of JSY in the state. The review of other documents mentioned earlier helped to triangulate the study outcomes.

#### FGDs

Nineteen FGDs were conducted among 141 beneficiaries of JSY during the six months preceding the study. There were nine FGDs for 78 ASHAs. Discussions with mothers explored their understanding of JSY and its impact on rationalizing maternal healthcare choice, their approach towards skilled birth attendance, maternal healthcare seeking, OOPS and financial risk-protection. Discussions with ASHAs provided the information on their understanding of JSY, payment mechanisms, performance motivations, challenges on effective functioning of JSY and adequacy of financial risk-protection for mothers.

#### Key informant interviews

Seven state, district and sub-district level MoH officials were interviewed on the functioning of JSY, challenges on its effective functioning and adequacy of financial risk-protection for mothers and financial incentives for ASHAs. The selected MoH officials were responsible for planning, implementation and monitoring of maternal and child health programs in the state.

### Data analysis

Verbal informed consent was obtained from each respondent after describing the study objectives and the use of forthcoming information. There was no refusal or withdrawal from any of the participants approached for the study. Information was collected by locally-based researchers through pre-tested and semi-structured guides in the local language *Odia*. The group discussions and interviews lasted between 30 and 45 minutes. Responses were audio-recorded, transcribed verbatim and translated to English. Data collection, coding and translation were supervised by two graduate researchers.

Emerging themes among the responses were coded, collated and analyzed by the first author to generate higher order generalizations through NVivo software. The thematic analysis of the responses was based on six categories; *influence of JSY on the place of delivery; JSY’s impact on OOPs; maternal healthcare seeking; performance motivations of ASHAs; determinants and outcomes of maternal healthcare cost; and the need to link JSY with other financial risk -protection measures.* The data on maternal healthcare utilization from HMIS were entered into Microsoft Excel spreadsheet and its trends were analyzed in STATA software. Ethical approval was obtained from an Independent Ethics Committee in Bhubaneswar. It consisted of stakeholders from the Ministry of Health and Family Welfare, academia, civil society organizations and the community.

## Results

### Trend of institutional deliveries vis-à-vis JSY benefits in Orissa

**Figure 1:**
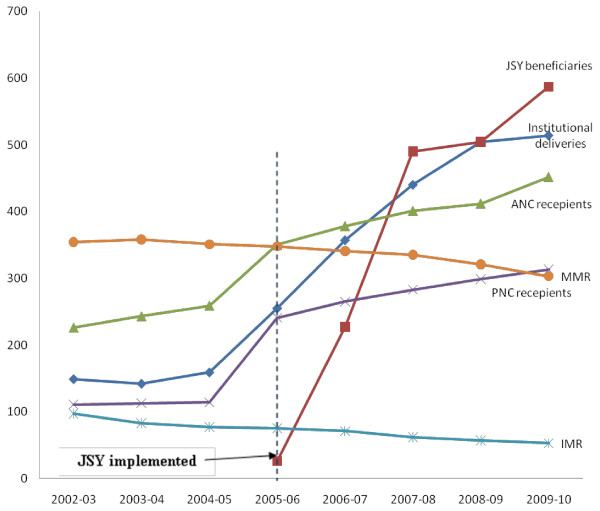
Maternal healthcare trend in Orissa before and during JSY.

During the JSY period (2005–2010), there has been a considerable improvement in maternal healthcare utilization (Table 1). The number of institutional deliveries grew at 20.3% annually (255,323 to 514,792). The number of pregnant women receiving ANC and PNC increased respectively at 8.4% (350,982 to 452,980) and 5.9% (241,980 to 313,456). During the pre-JSY period (2002–2004), institutional deliveries, ANC and PNC grew at 2.2% (149,341 to159,126), 4.8% (226,489 to259,376) and 0.9% (111, 257 to 241,980) respectively. Thus, JSY’s possible positive net contribution to the spread of institutional deliveries, ANC and PNC was 18.1%, 3.6% and 5.0% respectively (without controlling for other contributing factors). The proportion of deliveries receiving JSY benefits increased from 3.7% (26,407) in 2005–06 to 87.3% (587,158) in 2009–10. About 12% (70,458 out of 587,158) of JSY beneficiaries had domiciliary childbirth in 2010. The C-section rate among the JSY beneficiaries was 3.8% (22,312). The proportionate increment in ANC and PNC were lesser than that of institutional deliveries during the JSY period (Figure 1).

**Table 1:** Key maternal healthcare indicators pre- and during JSY in Orissa

**Year**	Total No. of deliveries*	**JSY beneficiaries**	**Institutional deliveries**	**At least one ANC**	**At least one PNC**	**Infant mortality rate (per 1000 live births)**	**Maternal mortality ratio(per 100000 live births)**
2002-03	811,104	Not applicable	149,341(18.4)	226,489 (27.9)	111, 257 (13.7)	97	354
2003-04	789,215	Not applicable	142,825(18.9)	243,125 (30.8)	113, 242 (14.3)	83	358
2004-05	743,711	Not applicable	159,126 (21.3)	259,376 (35.1)	114,231 (15.4)	77	351
2005-06	709,829	26,407 (1.9)	255,323 (36.0)	350,982 (49.5)	241,980 (34.1)	75	348
2006-07	772,736	227,204 (29.4)	357661 (46.3)	378,902 (49.0)	265,324 (37.4)	71	341
2007-08	701,215	490,657 (70.0)	440,234 (62.8)	401,196 (57.2)	283,556 (40.4)	62	335
2008-09	711,501	506,879 (71.2)	504,823 (71.0)	412,910 (58.0)	299,891 (42.2)	57	321
2009-10	672,585	587,158 (87.3)	514,792 (76.5)	452,980 (67.3)	313,456 (46.6)	53	303

Source: Health Management Information System Government of Orissa.

* Figures in parentheses are percentages to total number of deliveries.

### Experience and perceptions on JSY

#### About the JSY beneficiaries

Among the 141 discussant mothers, about a half did not have any formal education and 59.7%, (n = 84) were below the poverty line. There were 65.3% (n = 92) from socially backward communities and 36.3% (n = 51) in 15–23 age group. Some had first time child birth (23.9%, n = 33), while 44% (n = 62) and 32% (n = 45) were second-and third -time mothers respectively. Around 9% (n = 14) were second-time JSY beneficiaries, 10% (n = 14) had domiciliary child birth and none had adverse birth outcomes. There were 40% (25 out of 62) and 38% (17 out of 45) of second-time and third-time mothers respectively, who had institutional delivery without JSY benefits for the previous childbirth. Key background characteristics of mothers, ASHAs and MoH officials are given in Table 2.

**Table 2:** Background characteristics of the study participants

**Characteristics**	**No of participants**	**Mean age (range)**	**Mean years of schooling (range)**	**Social community (backward/forward)**	**Poverty status (BPL/APL)**
**Focus group discussions**					
Mothers groups (n = 17)	141	24(15–35)	02(0–9)	92/49	84/57
ASHA’s groups (n = 11)	78	32(25–40)	08(6–9)	52/26	56/22
**Key-informant interviews**					
MoH officials	07	33(24–51)	15(14–20)	2/5	0/5

### Influence of JSY on the place of delivery

According to the discussant mothers (93%) financial incentives motivated them and their households to opt for institutional deliveries. Both ASHAs (98%) and MoH (96%) officials also acknowledged JSY’s potential to induce institutional deliveries. One-third of the mothers would have delivered at home in the absence of financial incentives. However, young, literate and first-time mothers would have still had in-facility delivery, particularly at government health centers, even without the incentives. This preference was due to better facilities in accredited health centers and the current non-availability of birth attendants in the community. Preference for institutional delivery was the least among those aged above 25 years and staying far away from secondary level hospitals. While validating these revelations from the secondary sources, we found that there is an increasing trend for skilled birth attendance in the state [18]. Further, being far away from secondary hospitals reduce the probability of in-facility delivery [19].

*I went to a government hospital for delivery as ASHA didi (sister) told me about the JSY incentives.* [Mother, Nayagarh]

*Many mothers had hospitalized delivery due to JSY* [ASHA, Mayurbhanj]

*I feel a drastic improvement in institutionalized delivery with JSY program* [Medical Officer, Mayurbhanj]

### Impact on out-of-pocket spending (OOPS)

Many mothers (96%) were concerned about them incurring huge OOPS on maternal care. The average cost of pregnancy and delivery care in rural Orissa was about US$ 110 (US$ 70 in urban areas). The JSY incentive was able to cover only 25.5% (14.3% in urban areas) of this cost. Rural mothers had to mobilize OOPS for the rest US$ 80 (US$ 48 in urban areas). Many households approached pregnancy as a family event and mobilized resources on behalf of the women. Some women themselves looked for resources to finance their cost of child birth. Since the cost often exceeded the households’ ability-to-pay, they covered it through some distress coping measures such as unorganized loans and sale of hard-earned assets. Since the JSY incentive was received after the delivery, it could not prevent the need for raising short-term loans. After receiving the reimbursement, they were able to pay back at least a portion of their loans. A household survey in the state also observed that around 25% of households mobilized resources for maternal care through hardship means [20].

*I spent at least one year’s household income on pregnancy care.* [Mother, Gajapati]

*Our major constraint is that we do not have liquid cash with us when we need.* [Mother, Nayagarh]

**Figure 2:**
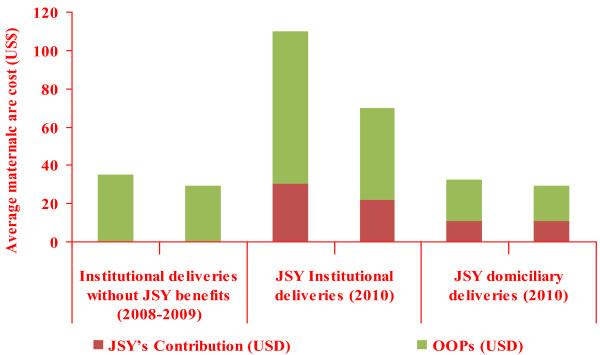
Share of JSY and out-of-pocket expenditure (OOP) in total cost of maternal healthcare.

A comparison of OOPS demonstrates that it was higher for institutional deliveries supported by JSY than domiciliary deliveries and non-JSY supported institutional deliveries (Figure 2). For instance, OOPS for mothers with domiciliary delivery was US$32 and US$29 in rural and urban areas respectively. It was US$35 for some rural mothers (US$ 29 in urban areas) for their previous institutionalized child birth during the last two years without JSY support.

### Maternal healthcare cost - decomposition, determinants and outcomes

A decomposition of the reported cost highlights that around 45% (US$45.5/110 in rural and US$30/70 in urban areas) was incurred during the ANC period and the rest during the delivery. A few had to spend 5% of their total cost on PNC. Even though the public healthcare system provided free ANC and PNC, women largely incurred OOPS in this regard. Some public providers directed many women to purchase vitamin syrups from private pharmacies. They also prescribed vitamin syrups (some upon demand by mothers), each costing US$ 3–5. The reported cost of care was 12.3% higher in private institutions (ANC 8.1%, delivery 4.2%) than in public institutions. The largest cost difference was observed on diagnosis (12% to 18%) followed by consultation fee (11.1% to 16%). Other sources also indicated that the cost of care on maternal care in Orissa was more in the private sector (Rs.3487), than that of the public sector (Rs.1494) [13].

*I went to the private clinic for ANC and it costed me around half of my total pregnancy related expenses*. [Mother, Mayurbhanj]

*My ANC was in a public hospital, but I purchased vitamin syrups costing more than Rs.120 (US$2.58) from a medicine store upon my request*. [Mother, Gajapati]

Another dimension of OOPS was on medicines and supplies during the time of delivery in public hospitals. The MoH officials were concerned about these additional expenses, though they expressed helplessness to overcome such system constraints. Some women expressed the inevitability to spend on transportation and it was more than what they received as incentive. These huge transport costs occurred in order to commute to an accredited facility despite the availability of facilities nearby. In the absence of incentives, women would have preferred to go to those non-accredited hospitals as they had faith on the providers over there.

*Many women have to spend on sutures, antibiotics etc. But, we are not able to do anything to overcome this.* [Medical Officer, Mayurbhanj]

*I spent Rs.300 (US$6.45) on a truck to go to the hospital where JSY benefits were available and it was more than what I have received.* [Mother, Gajapati]

About 40% of the JSY incentive was given back to the providers as ‘informal payments’, particularly in, but not limited to the public sector. Some mothers were concerned about ASHAs asking for incentives without providing sufficient support. In short, the financial incentives could not prevent irrational prescription practices, OOPS on supplies during delivery, and informal payments.

*Some providers at the primary health center behaved as if I was accepting their mercy and they were giving me some benefits through JSY incentives.* [Mother, Mayurbhanj]

*I paid more this time to the primary health center staff than my first delivery, when I did not receive JSY benefits.* [Mother, Gajapati]

*ASHA didi told me to report that she had escorted me to the hospital. But I was surprised, because she did not do so.* [Mother, Mayurbhanj]

*ASHA didi asked me a share on transportation reimbursement, though what I received was lesser than my actual spending.* [Mother, Nayagarh]

### Effect on maternal healthcare awareness, healthcare consumption and health seeking

The majority of women (irrespective of age and education) were not aware of the appropriate maternal healthcare requirements. They preferred institutional delivery or skilled birth attendance to comprehensive maternal healthcare. Only 23% (32 out of 141) had at least two ANC and PNC visits. Among the domiciliary deliveries with JSY benefits, three-fourth received at least one ANC. Women did not perceive maternal care or its entitlement as a right nor had adequate awareness on the whole objectives of JSY. Though public institutions catered to over 75% (95 out of 127) of the institutional deliveries, private institutions serviced over 65% (94 out of 141) of ANC.

Women did not differentiate the quality of care they received between the public and private hospitals as both had different merits and demerits of their own. They were more appreciative of the staff behavior and the status of supplies at the private hospitals. On the contrary, they felt the infrastructure status and drug efficacy to be the same in both the sectors.

*I think JSY aims at hospitalized delivery, as it is safer, if any complication arises during delivery.* [Mother, Nayagarh]

*I only prefer ante natal care as the child will not have any complications even if post natal care is omitted.* [Mother, Mayurbhanj]

*I did not have post natal care, as the chances of complications were less after delivery.* [Mother, Gajapati]

*I am happy that the Government at least gives us an opportunity to have institutionalized delivery. So, we should be obliged to adhere to the norms of JSY.* [Mother, Nayagarh]

### Performance motivations of community health workers (or ASHAs)

The CHWs, on an average spent five to six days in hospitals when mothers required C-sections. Otherwise, they spent 40 to 60 hours (60–80 hours in remote areas) for each mother during the entire maternity period. Linking remuneration with the conduct of each activity motivated ASHAs on their designated duties. ASHAs were satisfied on mothers’ attitude towards them. However, supply-side constraints such as lack of transportation and non-timely availability of JSY cards de-motivated their performance, besides affecting community’s confidence on them. In addition to their time, ASHAs also spent money on mothers’ food and medicines limiting their actual incentive to US$ 2–4 instead of the earmarked US$ 7.50. Despite this, ASHAs were proud of their moral responsibilities on supporting mothers. In order to serve the mothers better, they desired to receive more on-the-job training, supportive supervision and systematic capacity development.

*I am forced to perform each activity, otherwise I will not get the provisioned incentives*. [ASHA, Gajapati]

*How can we be heartless when we deal with pregnant mothers, who are our own sisters from the village?* [ASHA Mayurbhanj]

*I do spend from my purse on mother’s food and medicines at hospitals, but I often get lesser than what I spend.* [ASHA, Nayagarh]

*My knowledge and performance will be improved with further training on work and capacity development activities. I also need regular supervision and on-the-job support.* [ASHA, Mayurbhanj]

### Need to link JSY with other financial risk -protection measures

Women were largely concerned about OOPS on maternal healthcare. Yet, they were happy to receive JSY incentive as it reduced their financial burden to some extent. They were more appreciative of the ‘direct’ financial incentives under JSY than the ‘indirect’ financial risk- protection measures offered by other alternative health financing schemes. In one of the districts, the study had specifically explored about the *Rashtriya Swasthya Bima Yojana* (RSBY), a government scheme providing health insurance coverage for the poor [21]. Those who received both RSBY and JSY benefits were appreciative of their combined role in reducing OOPS. Among the three options such as free care, JSY alone, and a combination of JSY and other measures (e.g. RSBY), women preferred the third option. This preference was mainly due to their experience of incurring fresh OOPS through incentives such as JSY. Further, they had also perceived that the free care option too could induce OOPS of some kind.

*I like to receive JSY benefits as it is an incentive for us, if we deliver in a hospital.* [Mother, Gajapati]

*I want to have both JSY and any other scheme, because JSY is a financial incentive and other measures might help us to reduce instant spending from our side.* [Mother, Mayurbhanj]

*I am not able to believe a situation of free care. We every time have to spend on many things. People say that government hospitals are meant for poor people, but we never get absolutely free care.* [Mother, Gajapati]

ASHAs did not support integrating various incentive schemes (e.g. RSBY and JSY) since they were skeptical about the reporting and monitoring procedures. They preferred separate activity-based incentives under both schemes. MoH officials appreciated combining demand- and supply-side measures to meet the increased demand brought in by demand-side boosters. However, they were cynical on integrating JSY with other measures as it might negate the gains achieved so far on institutionalizing childbirths. They wanted to enhance ASHA’s incentives, but were against providing ASHAs a permanent employment as it could reduce their performance motivation. They urged for enhancing mother’s financial incentives to meet the additional expenses incur during hospitalization.

*I find it difficult to understand how the reporting and monitoring will be if we integrate different incentive schemes for mothers.* [CHW, Mayurbhanj]

*I prefer to have activity-based incentives under each scheme than combining the incentives.* [CHW, Gajapati]

*JSY could uptake institutional delivery; an integration of different schemes might undermine the role of JSY*. [Medical Officer, Gajapati]

*It is wise to increase the financial incentives of both mothers and ASHAs. But, giving a permanent cadre to ASHAs might reduce their performance motivations.* [Medical Officer, Nayagarh]

## Discussion

Demand side financing through the JSY scheme could enhance access to and utilization of maternal healthcare services. JSY’s contribution was evident in increased skilled birth attendance, ANC, PNC, and a partial financial risk-protection. The gains in institutionalization of deliveries are far greater than those of ANC and PNC, indicating the limited role of JSY in comprehensively addressing the maternal care needs. This could be due to the linking of entire incentives with in-facility delivery or skilled birth attendance than individually for each aspect of maternal care.

For a sustained improvement in maternal and child health, a comprehensive maternal care integrating its multiple aspects (e.g. health education, nutrition, ANC and PNC) will be required [22]. The non-encouraging trend of maternal deaths compared to infant deaths also highlights the relevance of post-partum care, as substantial share of maternal deaths might occur around the post-partum period [22,23]. Since DSF can motivate behavior change, their initiation, design, roll-out and evaluation need to be carefully planned to induce appropriate and rational health-seeking behaviors [4].

### Demand creation and supply strengthening

DSF is a way to translate healthcare needs into demand for health services [5]. In the presence of adequeate capacity, creation of additional demand can ensure effective service utilization [24].On the contrary, if there are supply constraints, quality of care might be compromised and further, providers and consumers may not be motivated on rational behaviors [8]. There are examples from Nepal demonstrating that private sector was excessively utilized as the public system faced systemic limitations [8]. Under the JSY scheme, the synergy between the demand-and supply-sides is enhanced through the intermediary or link-worker role of ASHAs. ASHAs also tried to neutralize the supply constraints by externally purchasing the supplies, which were unavailable at facilities for mothers during childbirth. However, this is not a sustainable solution as it might affect their performance motivation in the long run. Hence, supply-side strengthening is a necessary precondition for any demand-side incentive to produce desirable results.

### Is demand side incentive an appropriate financial risk-protection mechanism for maternal care?

A DSF scheme like JSY is not designed to pool financial risks [24]. While inducing a specific behavior change, it can trigger fresh OOPS and deepen household financial crisis. The financial catastrophe arises when financial access or risk-protection is not ensured otherwise for those who are exposed to the first-time institutionalized care and those who do not have adequate purchasing power. It is worth noting that JSY-supported institutional deliveries incurred more OOPS than domiciliary child birth and non-JSY supported institutional deliveries. If fresh OOPS fetch substantial health benefits and are not deepening the financial crisis of the payer, they might be justifiable. However, in this study, we do not have evidence in this regard to justify the presence of fresh OOPS.

The extent of OOPS largely depends on the design of the incentives, conditionality, provider and consumer accountability and service delivery status [25]. For instance, the use of a designated health facility was a JSY requirement. Though many non-designated facilities were available nearby, women incurred considerable OOPS to reach out to far off designated health facilities. Further, mothers and ASHAs spent privately on supplies due to systemic constraints. The consumer accountability also mattered as without realizing the real benefits of institutionalized childbirth, mothers preferred it over home delivery. This preference was owing to their realization that in-facility deliveries carried more financial incentives than home-based skilled-birth attendance.

Another concern was the lack of provider accountability leading to substantial OOPS in terms of informal payments. One of the reasons behind this perverse behavior could be lack of adequate incentives for them [25]. Many Latin American and Turkish DSF initiatives also had reported that adequate provider incentives were essential to ensure optimum provider accountability [25].Without proper provider behavior, it will be difficult to inculcate necessary consumer awareness and a sense of entitlement.

Financial incentives induce a particular behavioral change and improve awareness on health seeking. Having gained awareness, people’s compliance to the changed behavior is likely to be higher for discrete healthcare choices, especially for child birth (more of a household event) [6]. Therefore, there would be fairly increased demand for skilled birth attendance in future. However, it should be ensured that financial incentives for behavior changes are not inducing deeper OOPS and financial catastrophe. If women face substantial transport costs and informal payments, it may be wise considering cost-effective and safe home-based deliveries with skilled birth attendance [26,27]. The considerable presence of home deliveries among the respondents justifies this transportation constraints. Many home-based maternal care models exist and JSY can promote some of them [27,28].

### Harmonization of financial assistance measures

Many Latin American countries could demonstrat that financial incentives can be full-fledged and substantial to comprehensively cover various health aspects under a particular health goal (e.g. MDG 5) [28]. For JSY, it might be essential to integrate with other financial risk-protection or social assistance measures as Brazil and Nicaragua had demonstrated [29,30]. For instance, integration with health insurance might enable covering with careful planning the hospital costs fairly and improve consumer choices and provider accountability [25]. However, a federal structure like India with multiple ministries handling social assistance has to ensure a unified coordination [29]. A social assistance approach may enable DSF to uphold more sense of ‘right to maternal healthcare’ than a mere tight and conditional approach [31,32]. Currently, one Indian initiative namely *Muthulakshmi Reddy Scheme* comprehensively addresses ‘maternal care’ by incorporating nutritional aspects. However, the provision of cash transfer after childbirth makes this scheme unfit to track each outcome [33].A provision of fixed sum or pumping money, rather than objective based transfer to women may not achieve the expected results. This is certainly because of the households’ alternative needs, prioritization and gender power structure [34,35].

### Community health worker model for incentivized maternal healthcare

India brings in an encouraging model on community health workers as grass roots level change makers for healthcare. The optimum level of compensation for such a voluntary cadre is still debatable. We observed that the volunteers with activity-based financial incentives and service delivery improvements can enhance community health awareness and service utilization. However, maintaining optimum performance motivation for such volunteers especially through adequate remuneration, supervision, capacity development and monitoring is an emerging need [36,37]. This requirement was evident in a fewer cases where ASHAs demanded for remuneration without real performances. As pointed out by the stakeholders, their performance might be less-optimal, if given a permanent cadre or being asked to perform non-incentivized activities. A democratic revision of their incentives may be worth maintaining their motivations. While fixing up incentives, the time spent on processes also needs to considered than the end activity alone.

### Strengths and limitations of the study

This was one of the unique attempts to look at the financial risk-protection and financial access to healthcare under demand side financing. As applicable to the qualitative research methods, our study might not have wider implications beyond the study context. Though we looked at the secular trend of maternal healthcare consumption, the study design did not allow us to consider the factors attributable to it other than JSY. There was scope for recall bias while exploring the OOPs on child birth without JSY benefits. However, considering the universality of the program and its extent, the findings add to the rare global evidence base on DSFs. The recommendations might carry special value for the design and implementation of DSFs in similar LMIC settings. We also validated the healthcare costs and financial catastrophe on maternal care from other sources in the study settings.

## Conclusion

The Indian version of the DSF incentive appears to have enhanced financial access to and utilization of maternal healthcare, particularly institutional deliveries. The presence of financial risk-protection in JSY-supported childbirth was partial. It did not adequately link institutional delivery with ANC and PNC. Similarly, the knowledge transfer on maternal healthcare was limited and lopsided due to which fresh out-of-pocket spending was triggered by this incentive. An integration of JSY with similar social or financial risk-protection measures is likely to provide comprehensive financial risk- protection. Such an integrated approach would also enable addressing maternal health beyond ‘maternal healthcare,’ upholding awareness on maternal health entitlements and ensuring sustainability through pooled resources.

## Competing interests

 The views expressed in this paper are solely those of the authors and not of their organizations.

## Authors’ contributions

SSG and DV conceptualized the study, designed the study tools and drafted the manuscript. SSG drafted the first version of the manuscript and analyzed the data. Both authors read and approved the manuscript.
